# Abnormal Respiratory Sounds Classification Using Deep CNN Through Artificial Noise Addition

**DOI:** 10.3389/fmed.2021.714811

**Published:** 2021-11-17

**Authors:** Rizwana Zulfiqar, Fiaz Majeed, Rizwana Irfan, Hafiz Tayyab Rauf, Elhadj Benkhelifa, Abdelkader Nasreddine Belkacem

**Affiliations:** ^1^Faculty of Computing and Information Technology, University of Gujrat, Gujrat, Pakistan; ^2^Department of Information Technology, College of Computing and Information Technology at Khulais, University of Jeddah, Jeddah, Saudi Arabia; ^3^Independent Researcher, Bradford, United Kingdom; ^4^Cloud Computing and Applications Reseach Lab, Staffordshire University, Stoke-on-Trent, United Kingdom; ^5^Department of Computer and Network Engineering, College of Information Technology, UAE University, Al Ain, United Arab Emirates

**Keywords:** respiratory sounds, abnormal respiratory sounds, continuous adventitious sounds (CAS), discontinuous adventitious sounds (DAS), deep CNN

## Abstract

Respiratory sound (RS) attributes and their analyses structure a fundamental piece of pneumonic pathology, and it gives symptomatic data regarding a patient's lung. A couple of decades back, doctors depended on their hearing to distinguish symptomatic signs in lung audios by utilizing the typical stethoscope, which is usually considered a cheap and secure method for examining the patients. Lung disease is the third most ordinary cause of death worldwide, so; it is essential to classify the RS abnormality accurately to overcome the death rate. In this research, we have applied Fourier analysis for the visual inspection of abnormal respiratory sounds. Spectrum analysis was done through Artificial Noise Addition (ANA) in conjunction with different deep convolutional neural networks (CNN) to classify the seven abnormal respiratory sounds—both continuous (CAS) and discontinuous (DAS). The proposed framework contains an adaptive mechanism of adding a similar type of noise to unhealthy respiratory sounds. ANA makes sound features enough reach to be identified more accurately than the respiratory sounds without ANA. The obtained results using the proposed framework are superior to previous techniques since we simultaneously considered the seven different abnormal respiratory sound classes.

## 1. Introduction

Respiratory sound (RS) attributes and their analyses structure a fundamental piece of pneumonic pathology such as COVID-19 pneumonia, and it gives symptomatic data about a patient's lung. Lung sound is produced when air flows during the process of respiration. A couple of decades back, doctors depended on their hearing to distinguish symptomatic signs in lung audios through utilizing the standard stethoscope equipment. The typical stethoscope is usually considered a cheap and secure method for examining the patients, other than setting aside less effort required for the conclusion. Furthermore, it gives much data about the respiratory organ and the indications of the sicknesses that influence it (1, 2).

Later on, the auscultation by stethoscope is whimsical because it relies upon the doctor's capacity and the low affectability of the human ear hearing. However, non-stationary signs are hard to examine and challenging to recognize if not done by a well-prepared doctor; this may prompt wrong analysis. As of late, with the guide of electronic stethoscopes combined with pattern recognition and artificial intelligence, the mechanized respiratory sound examination has drawn much consideration since it conquers the confinements of normal auscultation and gives an effective technique to clinical conclusion (3). Machine Learning (4) and Deep learning approaches play an essential role in health care (5) and industrial applications (6, 7) for prediction and optimization.

### 1.1. Types of Respiratory Sounds

Since the classification criteria for respiratory sounds was defined in the 10th International Lung Sounds Association (ILSA) Conference, the respiratory audios classification step by step has become the focal point of audio respiratory examination. Respiratory sounds are partitioned into two different categories (normal and abnormal).

Normal respiratory sounds are those when a patient has no respiratory issue. Meanwhile, unordinary sounds when a patient is suffering from respiratory problems (8). As we talk about their further subtypes, they have “tracheal,” “bronchial,” and “broncho-vesicular” sounds. Audio of normal respiration is described through a commotion in the process of inspiration. Scarcely discernible clamor is in the process of termination/ expiration. In rough artery like “tracheal,” ordinary audios of respiration described through wide range noise, for example, the clamor has multiple parts of higher-frequency, these are capable of being heard in the process of inspiratory and expiratory period (1).

On the other hand, the second category of respiratory sounds is abnormal respiratory sound. They are different from the first one based on their natural and unique patterns in their behaviors. They appear when the patient has some respiratory issue and suffering from respiratory problem (8).

### 1.2. Subclasses of Abnormal Respiratory Sounds

Abnormal sounds are unwanted respiratory sounds that are excessively forced on ordinary breath sounds. Abnormal sounds have low power or strength during respiration. These sounds are classified based on some factors that help in the detection of each class separately. Unusual sounds are broadly categorized as continuous and discontinuous sounds, and that discrimination is due to the variance in their duration of occurrence while breathing (9). Continuous Adventitious Sounds are fallen in the category of unusual sounds, most of the time having 250 ms, but it is not valid for all the CAS, e.g., rhonchi. Based on the pitch, these sounds are classified as high pitch (Wheeze and Stridor)and low pitch (Rhonchi and Squawk) sound. On the other hand, Discontinuous Adventitious Sounds also fall in abnormal sounds, just like CAS. DAS is <25 ms in duration and is further categorized as fine crackle, coarse crackle, and pleural rub.

#### 1.2.1. Wheeze

Wheezes are sharp, regular, and constant extrinsic audios having a pitch with a minimum of 400 Hz. They are usually caused by airway narrowing, which then causes an airflow limitation. Wheeze sounds do not necessarily have durations of more than 250 ms. Some have reported that Wheeze can have minimum durations of around 80–100 ms (10). Diseases associated with Wheeze are asthma and COPD. If the Wheeze is localized, it may be caused by a foreign body blocking the airway, like a tumor (11).

#### 1.2.2. Rhonchi

Rhonchi are low pitched and musical sounds of unusual RS. Rhonchi encountered continuous adventitious sounds, and they can be heard during the inspiration phase, mainly in expiration, or during both phases of respiration (8). They described a predominant frequency range of around 200 Hz with durations of around 80–100 and usually nonstop in nature. Rhonchi are observed on both phases (inspiration and expiration) and caused by airway thickness in large section (12).

#### 1.2.3. Stridor

Stridorsounds are classified as hilarious inspiratory sounds, and it is a type of CAS. Stridor sounds are produced in the larynx or bronchial tree by turbulent airflow and are similar to airway obstruction (upper). They have their presence at the inspiration phase due to narrowing the upper airway, and it can be heard on expiration or even in both phases. Stridor has a pitch of more than 500 Hz and a duration of more than 250 ms. They are usually louder and harsher than wheeze sounds. The diseases associated with Stridor are epiglottitis, croup, and laryngeal edema because every disease is related to airway obstruction (13).

#### 1.2.4. Squawk

Squawk sounds to DAS usually have short durations and are hearable at the phase of inspiration. These sounds are low in pitch (like wheezing sounds), and their frequency ranges from 200 to 300 Hz (8). Squawk observed when patient suffering from hypersensitive pneumonia or common pneumonia (14). Fine Crackle: Crackle sounds are the dangerous extrinsic sound of respiration. Crackles are irregular in their behavior patterns, described by explicit waveform, span, and position of sounds in the respiratory cycle. Crackles have two attributes and are portrayed through their span as “fine pops/ crackle” and “coarse pops.” Explosive openings of the small airways cause fine crackle sounds. They have a little span (around 5 ms) with high frequencies (650 Hz). Fine crackles can be heard on the phase of inspiration. They usually cause the diseases like pneumonia, congestive heart failure, and lung fibrosis (12).

#### 1.2.5. Coarse Crackle

Air bubbles generate coarse crackle sounds in large bronchi. The Coarsesounds are audible when inspiration is in its early phase/stage and hearable at the expiration phase. “Coarse pops/ crackle” are low pitch, around 350 Hz, and have a long span (about 15 ms). Coarse crackle sounds can be heard in patients with chronic bronchitis, bronchiectasis, as well as COPD (15).

#### 1.2.6. Pleural Rub

Pleural rub is non-rhythmic and encounters in the category of DAS. When pleural aggravated surfaces rub each other in breathing, then “pleural rub” audio produced has a low pitch, generally below 350 Hz, and only appears for 15 ms. The PR sounds are produced by the friction and audible during both phases of expiration and inspiration. The pleural rub is usually caused by inflammation of the pleural membrane, and it can also cause pleural tumor (8).

In the field of bioinformatics, respiratory sounds classification (RSC) has become the center of attention. Researchers worked in the past and still working on RSC (normal and abnormal) to get precise results. As research goes on and on, researchers face many difficulties in RS classification. As we talk about RS's previous classification research outcomes, the main concern was the misclassification of RS (specifically between the subclasses of adventitious RS). This was because they were not distinguishing the sounds with minor variance (change) in their behavioral patterns and did not encounter all abnormal respiratory sounds simultaneously. The study's goal was muddling the functions of examination/ analysis and discrimination of ARS (those sounds showing minor differences in their frequencies and period) toward the appropriate classification of respiratory sound.

Respiratory sound examination/classification is a vital part of the Auscultation process. Doctors perform the auscultation process through typical techniques, leading them to wrong decisions due to the different dependencies. Research tells us that lung disease is the third most ordinary cause of death worldwide, so it is essential to classify the RS abnormality in an authentic way to overcome the death rate.

The proposed methodology based on Artificial Noise Addition technique (ANA) is used to enhance the features of ARS and helps in feature extraction through feature maps. Furthermore, “ANA” aid in differentiating the subclasses of abnormal/adventitious sounds more accurately. As far as we classified ARS using “ANA” phenomena, classification results boost, which ultimately smooth the doctor's progress flawlessly to carry out the auscultation process. It will support consultants in the auscultation process to identify the disorders or abnormalities related to the human respiratory system. When the doctors identify the disorder in the particular RS, they will diagnose the associated diseases caused by these sound abnormalities and prescribe the best treatment to a patient suffering from the syndrome.

The first thing is to focus on the number of classes of ARS. As per our knowledge, no such work with several lung sounds has been done so far; previous researchers classified few abnormal respiratory sounds, most common in patients' lungs that ultimately cause abnormality in them. So, we have incorporated the seven different classes of respiratory (lungs) sounds in our designed framework for the sake of classification.

The proposed framework based on the spectrogram of each respiratory sound by applying the Fourier transformation for the relative spectrogram of sound. Contribution for further enhancement after getting the required spectrograms, the framework makes the wave spectrogram more strengthen and clear through adding some artificial noise of the exact spectral nature. This process serves as constructive interference to augment/enhance the actual spectrogram of sounds. An “Artificial noise addition (ANA)” phenomenon helps prominent the pattern and aids feature extraction; make it more accessible and fair. “ANA” does not mean that any traditional background noise. Finally, feature extraction is done through feature maps on spectral data and different classifiers (VGG, ResNet, InceptionNet, and AlexNet) used for classification purposes.

The rest of our manuscript is organized as follows: related work is discussed in section 2. Section 3 describes the complete overview of the applied framework and the steps involved in its deployment. Experimental setup and results are discussed in section 4, while the conclusion and recommendations have been discussed in section 5.

## 2. Related Work

For normal and abnormal respiratory sound classification real-world dataset is designed to automate the process. By taking advantage of previous studies, researchers used “spectral” and “wavelet” techniques for feature extraction without enhancing the features of a particular sound (2). “Convolutional neural networks CNN,” “Hidden Markov Models HMM,” and “Gaussian Mixture Models GMM” were collaborated for decision level. Such a scheme could support the classification precision and outperform to distinguish respiratory sound with 66.7% precision. Other subclasses of respiratory sounds were not engaged, i.e., St, Sq, and Rh (16). As research tells us that lung disease is the third most ordinary cause of death worldwide, so it is important to classify the RS abnormality in a true way to overcome the death rate. Short-Time Fourier Transform (STFT) was used for feature extraction, and two other deep learning methods were used for classification from spectrogram after feature extraction. ICBHI database was put into consideration with different frequencies and noise. Two approaches were applied; the first used deep CNN for feature extraction and SVM for classification, whereas the second used a spectrogram. 65.5 and 63.09%, accuracies were recorded for first and second method respectively (17).

Chronic Obstructive Pulmonary Disease (COPD) and usual sound classification depend upon respiratory audios using different machine learning approaches. Twenty-five normal and thirty COPD sounds are collected and analyzed. Thirty-one features of audios were extracted and evaluated. COPD breath audios are categorized by using “Support vector machine (SVM),” “k-nearest neighbor (KNN),” “logistic regression (LR),” “decision tree,” and “discriminant analysis (DA)” algorithms. After the study's evaluation results, the linear predictive coefficient and median frequency are best for classification tasks with an accuracy of almost 100%—the feature extraction process done without making them more strengthen. The multi-centered dataset can reduce the accuracy rate if encountered. Although they got the highest accuracy in terms of classification, this approach only classifies “normal” and “COPD” objects in RS. Other subclasses/subtypes of abnormal respiratory sounds were not considered, i.e., wheeze and crackle (18). When we look upon the medical diagnostic solutions for abnormalities, it took a lot of time and cost; a cheap solution was proposed. Patient internal sounds were recorded by Stethoscope, lungs, and heart sounds. These readings proved much and more significant to assist the physicians. They determine the disease of the patient's lungs based on tagged audios through ML algorithms; through NN, they achieved the highest accuracy was 77.8%. However, only three types of sounds are considered (e.g., wheeze, bronchia). Other abnormal RS classes were not identified, and also accuracy can be affected due to the nature and size of the dataset, and multiple point classification is better to use (19).

A semi-supervised approach based on the graph is “one-class support vector machine (OCSVM),” which indicates the relationship between the entire sample and describes normal and abnormal lung sound (LS). “OCSVM” used a minimal amount of labeled samples for training purposes and tested the approach with a large number of unlabeled samples for testing purposes with an Accuracy of 60–80% by increasing the NL's. If a fair dataset is processed with this method, accuracy will decrease gradually because it supports artificial datasets. “OCSVM” was a detection technique for some classes of abnormal RS and was only suitable for the identification of ordinary, crackle, and wheeze sounds. The classification was not performed, and also, it had not encountered the other sounds, e.g., Stridor, Rhonchi, and squawk (20). Another method for LS classification introduced named “Multilevel Wavelet Packet Entropy (MWPE)” uses many entropy measurements. “MWPE” was a combination of two existing “Renyi” and “Tsallis” entropy. Feature extraction was done without making them more prominent. “MWPE” gained 97.98% accuracy during the classification of RS when decomposition levels are four (4) by using Shannon, whereas “MWPE” includes “Renyi entropy” and “Tsallis entropy” gained 93.94%, 57.58% accuracy, respectively. Five classes of RS were countered, i.e., normal, wheeze, crackle, stridor, and squawk. Discontinuous abnormal respiratory sound includes fine crackle, and coarse crackle was not considered (21).

In 2019, a Cancer diagnosis in its early growth stage was made with a 0.0212 error rate that assumed the least rate and 99.48% predictive rate recorded by applying soft Neural computing techniques like “Discrete AdaBoost optimized ensemble learning generalized neural networks.” Data were taken from the “ELVIRA” source, mixed with noise and other anomalies. Removal of noise and anomalies were done with the help of the normalizing smoothing technique. After preprocessing, feature selection and dimension reduction to reduce the complexity of data were made through the Wolf heuristic features technique. When it considers how many classes were successfully detected by “Discrete AdaBoost,” it only detects the normality and abnormality of a particular sound. The main focus of the “Discrete AdaBoost” was the detection of abnormality regardless of classification. Furthermore, subclasses of abnormal RS were not encountered. An optimized approach for capturing the data can have better outcomes if used in the future (22). A study was conducted in 2019 to find out the environmental factors that impact human health in many ways. Multiple types of data sources were involved, includes images of individuals and the information related to the social activities within a particular environment. Multiple techniques were introduced and texted to check the impact of environmental factors on human health. After characterizing many spatially correlated with deep convolutional neural networks, Deep CNN has a strong effect on the human health of an individual. Deep CNN has more potential to do a better job (23). A review was conducted to check the multiple techniques for extracting the feature and classifying respiratory sounds. They discuss NN, ANN, CNN, KNN, and many other algorithms for abnormal RS analysis. In the end, it has been stated that the “CNN” consider as the latest approach for implication (24).

Fifty to two-hundred Hz is the fundamental frequency range found for features that are inputted by “Hidden Markov models (HMM)” and “Gaussian mixture models (GMM)” when they are joined together in a hybrid form. 39.56 ranking gain from spectral subtraction for unwanted sounds (noise) removal from multiple sounds directories in preprocessing. Features are extracted by removing noise without making them dense or strengthen. They direct the researcher to work on advanced noise suppression techniques that will improve the overall score. As it has cleared that the researcher did not encounter the further subclasses of abnormal RS, i.e., stridor, squawk, pleural rub, and rhonchi, only wheeze and undefined crackles (mixed fine and coarse crackle sounds) were considered (25). The feature extraction (FE) technique was introduced in 2018 when researchers altered the traditional Grey Level Difference Matrix (GLDM) in a new form. Texture analysis (TA) mixed with “GLDM.” Texture analysis was done using signal ID or the value of a particular pixel. Performance checked through multilayer perceptron (MLP) in combination with SVM. 94.9% accuracy was recorded at sample distance d = 10. When the researcher used five features, “GLDM” and “cubic SVM.” Taking a look at several classes that were considered in research was normal, wheeze, faint crackle, and stridor, other sound classes like squawk, fine crackle, coarse crackle, and rhonchi were not involved (26). The respiratory sound classification was done in the presence of multiple types of noise (includes 3–4 classes, e.g., speaking, coughing, heart sounds, and many more). An algorithm was proposed for feature extraction, and that was a fresh nonlinear approach for better representation and discrimination of RS. Later on, 49.86% accuracy was recorded when applied to classify the multiple RS sounds having a variety of noise in them, as mentioned above. However, here is an issue, only four classes (normal, wheeze and crackles, and crackle plus wheeze) are considered, and the noise in sounds may confuse its nature with crackle sounds as well as the other abnormal RS classes were not considered, i.e., stridor, squawk, pleural rub, and rhonchi (27).

When researchers examined the asthmatic patients through ENS, SVM, and Spectral integrated (SI) features, they categorized patients based on their illness degree (first stage, moderate stage, or last stage). Fifty-five patient samples were taken for examination, and multiple Statistical analyses were performed to check the different patterns/manners of features with relentlessness stage of different groups. Overall best results gained by these methods for the first, moderate, and the last stage were 95, 88, and 90%, respectively. Still, that method identifies only the wheezing sounds (not other abnormal RS subclasses) and lacks focus on better representation of patterns, e.g., frequency and phase (28). An Overview of deep learning was done for radiology for disease detection, classification, quantification, and segmentation. Radiology is an intrinsically information-driven approach. It is beneficial for using information handling strategies. In this work, the Association of University Radiologists Radiology Research Alliance Task Force on Deep Learning reviews deep learning to the radiologist. The article introduces a review of deep learning in an understandable way to radiologists to analyze past, present, and future applications, just as to assess how radiologists may profit from this new tool. These researches portray a few regions inside radiology wherein deep learning methods have the most critical effect: malady recognition, classification, evaluation, and division (29).

COPD is a respiratory illness that is caused by smoking. Respiratory Database @TR was used to analyze the “COPD” patients. The second Order difference plot (SODP) method was introduced for analysis. Performance gain recorded 95.84% (accuracy), 93.34% (sensitivity), and 93.65% (specificity) when “Second-Order difference plot (SODP)” designed for examination levels of COPD sounds. Overall gain is high because SOPD is based on many factors related to sound's three-dimensional quantization. Deep belief networks (DBN) were used for classification and training purposes (30). Three approaches (Approach 1: KNN, SVM, and GMD, Approach 2: local binary pattern (LBP), and Approach 3: CNN designs) perform the critical task of analysis, recognition, and distinction of LS with 95.50% accuracy. The highest numbers of adventitious respiratory sounds classes were used in that method till now. Wheeze, fine crackle, coarse crackle, stridor, and squawk were involved; only pleural rub was compromised and not countered. However, the fact is, the researcher used a dataset of 7–8 sounds/recordings to classify the sounds, which is very small from a research perspective. More consideration is required for better pattern recognition, and this may be done when we involve spectrums of sounds and enhance their features through adding artificial noise in them for further research (1).

Another problem is when a doctor/specialist sees the ECG waves; there is the possibility of error in the vision of humans (doctor itself) during reading because of the petite/short wavelength, tiny span, and arbitrary/random phase shift of ECG signals. Convolution networks were used and tried to sort out the particular problem as mentioned above. Eventually, two approaches were adopted with multiple different factors to check the maximum accuracy rate of the problem. De-noising was involved in checking the difference of accuracies between noised and de-noised data. The first approach gained the 93.53% accuracy rate when CNN was applied on data with noise and the second one gained a 95.22% accuracy rate when ECG waves are free of noise (31).

Researchers designed a low-cost stethoscope for examining lung sounds and classify them into different classes. Two algorithm approaches were used to classify LS (MFCC & spectrogram). A method of “Mel Frequency Cepstral Coefficient (MFCC),” with the help of an image spectrogram, classifies the data of 17,930 sounds. The classification was the main objective of this work. CNN and the traditional SVM algorithm classify the four different breathing audio sounds. Normal, Rale, and Rhonchi audios were classified with different precision results. Other classes such as stridor, squawk, and pleural rub were not considered. As we talk about results, “MFCC” gain results in a range for four classes; first-class CNN 86%, SVM 86%, second class CNN 76%, SVM 75%, third class CNN 80%, SVM 80%, and last class have CNN 62%, SVM 62% accuracy rate, respectively (15). The machine learning approach was used in the next research, and the researcher classifies the normal and wheezing sounds from the data, which consist of 43 samples of recordings. They got minimum samples to detect wheezes; they did a data augmentation step on the sample for data enlargement. WD CNN architecture used minimal steps and took no time to preprocess the data to remove the anomalies. It is also an insensitive method to shifting lung sound, and noise observed externally from environmental factors. As a result, automatic wheeze detection in lung sounds through CNN Achieved an accuracy of 99%, but only Wheezing sounds are detected here. Low pitched sounds are not detected through this mechanism (32). For pulmonary issues detection, different methods and techniques are used to analyze the spectrum of RS. Fast Fourier Transform (FFT), AutoRegressive (AR), and the AutoRegressive Moving Average (ARMA) were used for calculating the densities of the spectrum of RS. Feature vectors were given as input to ANN. Spectrum analysis performance was recorded in classification accuracy (CA) as 85.67% for AR, 84.67% for ARMA, and 80.33% for FFT, but that accuracy is only for limited data points new or large test data inputted, then accuracy is not guaranteed. It only checks the normal and COPD individuals, not identifying the other abnormal RS classes such as wheeze, rhonchi, and stridor (33).

Lung sound classification is often done by applying the technique of signal processing (SP). Multiple “SP” methods were applied to RS/LS and classified them based on their nature and behavior. “Multi-scale Hjorth descriptor (MHD)” was introduced to classify RS in a particular class. “MHD” measured LS signal complexity. Signal complexity and accuracy were measured based on scale 1–5 and “Multi-scale Hjorth descriptor” measurements took with 96.06% accuracy, but the accuracy will not improve by enlarging the scale (34). Radiographs are usually very tiny in size; used for reliable classification of different most relevant images to medical science. Here the approach adapted where the techniques in computer visions and deep learning were bridged. It was a challenge for developers and researchers, which was tackle through deep-CC. The researcher divided the dataset into three groups for training, testing, and validation purposes. They applied the data augmentation technique for data enlargement as deep CC needs massive data for their processing. After experimentation, Deep Convolution Network (DCN) gained 97.73% accuracy when it is integrated with different GoogleNet and ImageNet (35). The following study examined the three different classes of respiratory sounds and evaluated them based on their distinctive nature and exclusive patterns. Experiments were conducted on thirty (30) dataset items with the help of an artificial neural network (ANN). “ANN” worked as a classifier in experimentation. Mel-frequency Cepstral coefficients (MFCC) pull out the statistical features from a dataset of RS. Further, the attributes for impure lung sound were acknowledged. They have discovered that their recently examined attributes were stronger than existing ones and showed better precision. Every single class of adventitious RS was not occupied here. The considered classes of RS were normal, wheeze, and faint crackle. CNN with wavelet-based features and MFCCs classify the sound and got an accuracy of 94.98, 97.83, and 97.6% for normal, wheeze, and crackle, respectively (36).

Pattern recognition classifies LS through “Genetic Algorithm (GA),” “Fisher's Discriminant Ratio (FDR)” was used to overcome the dimensionality of LS, and “Higher-order statistics (HOS)” used for feature extraction in this research. “k-nearest neighbor” algorithm used for pattern event recognition with an accuracy of 94.6% invalidation. Pattern recognition can be enhanced for more classes of respiratory sounds to improve classification performance. Four classes such as “normal,” “coarse crackle,” “fine crackle,” and “wheeze” were considered and recognized whereas, St, Sq, Rh, and PR were omitted (37). Identification of crackle in respiratory sound causes pulmonary abnormalities in the human body. PC has a minimal period and discontinuity, which usually appear at the inspiration and expiration phases. Multiple entropies did detect these PC. However, the “Tsallis entropy” was utilized as a feature extraction technique and achieved 95.35% accuracy for pulmonary crackle sound detection. The significant edge of using the Tsallis entropy was that it produces fewer features, but it took a minute dataset. Plus, it had not encountered all classes for abnormal sounds (just functional for faint crackle sound) (38).

Rational Dilation Wavelet Transform (RDWT) Discriminate the crackle, normal and wheeze sounds gained the accuracy of 95.17% for the total sound signal type. Q-factors were excluded in this approach because they cannot cope with the oscillatory signals (rapidly changing). Lung sounds were classified, and the feature was extracted without enhancing them, and while taking a look at several classes that were put in approach, were only three as mentioned above. The approach was suitable for high-pitched RS because low-pitch RS was not well-thought-out and performance reduced due to superimposed (cover-up) crackle and wheezes. So researchers still need a method and more consideration to tackle the issue if they overlapped to each other (39).

In 2015, automatic analysis of lung sound recordings (captured through electronic gadgets) involves and classification was done with the help of characteristics of LS signals. Feature extraction was done on a tool named “MATLAB” and classification through a combination of “artificial neural networks (ANN)” with “neuro-fuzzy inference systems (ANFIS).” The system successfully classified different RS/LS and gave 98.6% of accuracy. Results can be enhanced if correlation involves order, let alone the complexities in the feature extraction process (40). Pulmonary chaos/issues analysis based on auscultation to classify respiratory sound. The classification was done on normal and continuous RS includes Wh, Rh, St, and Sq. Discontinuous sounds like FC, CC, and PR were not put into the study. Features extraction was done on time-frequency based with an average set of frequencies. Spectro-temporal features extraction was used and got classification results for inspiratory and expiratory parts separately though “Support Vector Machine.” When SVM was applied on actual recordings, the accuracy recorded for inspiration is 97.7% and for expiration 98.8% (41).

The main objective of this study was to detect pneumonia inpatient which is already suffering in “COPD.” Almost 50 patients were examined in the study as an initial dataset. A hybrid approach was adopted to detect contemporaneous pneumonia. The approach was a combination of “principal component analysis (PCA)” and “probabilistic neural networks (PNNs).” Short-time Fourier transform (STFT) is used to extract features from the dataset. Dimensions were reduced through “PCA” and “PNN” used for training classifiers. Results show 72% of sensitivity and 81.8% of specificity on cross-validation (42). The empirical classification (EC) method was introduced for respiratory sound analysis. Undefined normal and abnormal respiratory sounds come across for classification purposes, excluding their subclasses. The principle of multi-scaling was applied for signal enhancement and helped feature extraction while combined to imprison the inconsistency of sound signal. The empirical classification was dependent upon the principle of multi-scaling dimension reduction to boost the actual signal. EC applied on 689 recorded segments and gained 98.34% for classification (43).

In their research work, the researcher presented the “pattern recognition (PR)” scheme for the classification of RS. As a dataset, they took only normal and wheezing sounds for classification. Post-pre-processing was applied to raw data to enhance the process of classification. Feature extraction schemes were evaluated and compared with each other includes FT, LP coding, and MFCC. “GMM” with “ANN” was applied, and they used a “threshold” value for differentiating the wheezing sounds from normal ones. Their experiment results showed better performance from previous work (44). Texture-based classification named “LAC” was done on discontinuous sounds, i.e., fine crackle, coarse crackle, and squawk, to capture the changes in pulmonary acoustic, which is helpful in pathology. De-noising was applied to remove the background noise effect from discontinuous sounds. Four lung sound databases were used in studies includes 25 cases with 365 sounds. LAC was so simple because it introduced “texture” analysis for DAS. As talk about results of this approach, three databases out of four successfully classify the FC< CC and SQ. 100% accuracy for FC-SQ, 99.62% accuracy for CC-SQ, and 99.77% accuracy for FC-CC-SQ achieved, but only three classes were captured for “LAC” texture-based classification (45).

The analysis was drawn for respiratory sounds RS to check the ongoing RS methods and research. The researcher reviewed different technological and therapeutic experiences. The analysis involved a range of trends and techniques used to gather respiratory sounds RS for involuntary recognition gear development. Current tools are based on “fuzzy system (FS),” “artificial intelligence (AI),” “genetic algorithms (GA),” and “artificial neural networks (ANN).” Finally, they tried to find out some gaps in previous trends to facilitate the researchers for further enhancement in this area of RS identification, recognition, classification, and tool development for their analysis (46). Furthermore, researchers introduced a new technique or method for lung audios analysis by combining the two previous frameworks to maximize results. ANN joint with wavelet transforms in this approach. The procedure was simple; with the help of wavelet transform, they divided the audio dataset of lungs into its multiple sub-spectrums and learn the features of sounds from those spectrums. Researchers built a standard 19-40-6 for this framework. 91.67% accuracy was recorded through distribution WC-ANN architecture, but all types of abnormal RS are not identified and considered here, e.g., fine crackles, coarse crackles. Moreover, only ANN is used here (47). A comparative study was conducted to analyze and built libraries for normal and pathological. Feature extraction for healthy and pathological recordings was done through autoregressive (AR) schemes. By “AR,” a Quadratic classifier and KNN (two classifiers) were designed and analyzed. The performance was evaluated on multiple models (48).

As we talk about the phenomena of “Artificial Noise Addition ANA” used for training neural networks. Training of ANN with noise injection done to avoid over-fitting of curves and in results this phenomena gives better outcomes for resolving the over-fitting issues (49). On the other hand, the problem arises with labeling features, a layered design that adapts the “Noise” as a positive factor, named “Noise adaptation layer.” Features enhanced by “noise adaptation layer” and correct labels which make the training process of NN easier (50). Also, to increase the resilience of NN, the ANA method was applied to the patterns of particular objects (51). Another research shows how the artificial noise adding to the signal (input) positively impacts NN performance. This technique improves the measurements that are needed for secret keys (52). The detail comparison and overview of exiting “ARS” classes are given in Table 1.

**Table 1:** Comparison and overview of exiting “ARS” classes.

**References**	**Abnormal respiratory sounds**								**Approach**	
	**Continuous adventitious sounds**				**Discontinuous adventitious sounds**					
	**Wh**	**Rh**	**St**	**Sq**	**UC**	**FC**	**CC**	**PR**	**De**	**Cl**
Ntalampiras (16)	✓									✓
Haider et al. (18)	✓									✓
Lang et al. (20)	✓				✓				✓	
Rizal et al. (21)	✓		✓		✓			✓		✓
Jakovljević and Lončar-Turukalo (25)	✓				✓					✓
Rizal et al. (26)										✓
Serbes et al. (27)	✓				✓					✓
Nabi et al. (28)	✓									✓
Altan et al. (30)	✓				✓					✓
Aykanat et al. (15)	✓	✓								✓
Bardou et al. (1)	✓		✓	✓		✓	✓			✓
Kochetov et al. (32)	✓								✓	
Göǧüş et al. (33)	✓				✓				✓	
Sengupta et al. (36)	✓				✓					✓
Naves et al. (37)	✓					✓	✓			✓
Rizal et al. (14, 34)						✓	✓		✓	
Ulukaya et al. (39)	✓				✓					✓
Rajkomar et al. (35)										✓
Jin et al. (41)	✓	✓							✓	
Xie et al. (43)										✓
Hadjileontiadis (45)				✓		✓	✓			✓
Bahoura (44)	✓									✓
Kandaswamy et al. (47)	✓		✓	✓						✓
Research methodology	✓	✓	✓	✓		✓	✓	✓		✓

## 3. Materials and Methods

In research methodology, the classification of adventitious respiratory sounds was done using the respiratory sound data set from multiple sources. Sequence and detail of all leading points are given below.

### 3.1. Preprocessing of Sounds

The practice begins with audio dataset loading—raw data taken from multiple sources and preprocessing. In preprocessing phase, all audio files were converted into “wav.” format. The motivation behind this conversion is to perform further modification on sound samples. Redundancies were also removed and bring the data in normalized form.

### 3.2. Sound Signaling

The variation in the air produces sound. As “RS” are produced during the respiration cycle of a human being. When these sounds have represented the variation concerning time (t), it forms a sound signal. The extraction of information from a complex sound was done by converting the sound into analog or digital signals form. RS conversion into its first spectrogram (waveform representation) was done through the sound signaling process. Here the RS were converted into sound waves/signals having the information about their amplitude and time. Each respiratory sound has a different period and amplitude as they belong to a different category and show their unique spectrogram, which discriminates them from one another in terms of their pattern and behavior. For a basic understanding, take a look at the following equations. Wave period (t), frequency (f) as following;


(1) $$\begin{array}{l} Frequency\left(f\right)=1/timeperiodorf=\frac{1}{t} \end{array}$$

(2) $$\begin{array}{l} Timeperiod=1/frequencyort=\frac{1}{f} \end{array}$$

Whereas, the velocity is defined as following;


(3) $$\begin{array}{l} Velocity=frequency*wavelengthorv=f\times \lambda \end{array}$$

Rearranging the equation (3);


(4) $$\begin{array}{l} Frequency\left(f\right)=velocity/wavelengthorf=\;\frac{v}{\lambda } \end{array}$$

From equation (2) and (4), we'll get the value of time period in terms of velocity and wavelength;


(5) $$\begin{array}{l} Timeperiod=wavelength/velocityort=\frac{\lambda }{v} \end{array}$$

Relationship between the (*t*), (*v*), (λ) and (*f*) defined as above.

### 3.3. Fourier Transform

Fourier transform (FT) is a mathematical transform that decomposes a function (often a function of time, or a signal) into its constituent frequencies, such as the expression of RS represents in terms of the frequencies. Fourier transformation was applied on data (Python 3.7 with scipy library) to generate the spectrograms (sounds into its waveform representation) for unhealthy sounds and visualize their behavior. The motivation was to capture the discriminative frequency characteristics of lung sounds for better representation of different classes. Two approaches are applied, named “positive” and “complete” Fourier transformations, which enabled us to get the information about the respiratory sound frequency and its proportion in a particular signal.

Complete Fast Fourier Transformation: Complete FFT count the double side frequencies for positive and negative values simultaneously.


(6) $$\begin{array}{l} F_{ft}=\frac{{f\left(t\right)e}^{os}+S\left({f\left(t\right)e}^{os}\right)}{\left|f_{one}\right|} \end{array}$$

Where *S*(f(t)*e*^os^) indicates the size of sound signal and |*f*_*one*_| represents the both side frequencies in one transform.

Positive Fast Fourier Transformation: Positive FFT count one side frequency of sound signal


(7) $$\begin{array}{l} P_{ft}=\frac{F_{ft}+S\left(F_{ft}\right)}{\left|N/2\right|} \end{array}$$

Where *S*(*F*_*ft*_) indicates the size of Fast Fourier Transformation and ||*N*/2|| represents the half frequency range for each input bit. We get the spectrogram of respiratory sound with relevant frequency (*f*) according to the time (*t*) and amplitude (*A*) of sound. FT is employed to increase the performance of the proposed system. Another mathematical representation of Fourier Transform in sine wave is described in Equation (8).


(8) $$\begin{array}{l} G\left(\omega \right)=\int_{-\infty }^{8}{\left({f\left(t\right)e}^{os}{f\left(t\right)e}^{as}\right)}^{--j\omega t}dt \end{array}$$

In above equation *f(t)* represents the input sound signal of RS and *G(*ω*)/ F(*ω*)* represent the fourier transform. Integral of Fourier transform is over −∞ < *t* < ∞. It's a time domain representation of input sound signal. Here is the input signal *f(t)* is multiplied with composite exponential function. The complex exponential function broken into component according “Euler's formula”:


(9) $$\begin{array}{l} \text{e}.\;--\text{j}\omega \text{t }=\text{ cos }\left(\omega \text{t}\right)\;+\text{ jsin }\left(\omega \text{t}\right)\; \end{array}$$

The similarity of input signal *f(t)* with complex exponential is described by a set of coefficients obtained through this equation. In other words, it tells how the input signal is similar to series of frequencies.

### 3.4. Spectrogram Robustness

Contribution for further enhancement, the spectrogram of RS with the gradual (minor) change, we added the artificial noise of exact spectral nature to enhance the actual spectrogram of faded sounds to make them more strengthen and enhance their robustness. As we applied the “noise addition” on respiratory sounds, neither the approach has changed the actual behavior of the spectrum nor produced any abnormality in the sound spectrogram. An “Artificial noise addition (ANA)” phenomenon only helped prominent the pattern and made feature extraction easy and fair. “ANA” does not mean that any traditional background noise in RS. References to “ANA” are given in the previous section of the literature review. The following equation does artificial noise addition.


(10) $$\begin{array}{l} F_{ft}^{Noise}=F_{ft}+F_{ft} \end{array}$$

**Figure 1:**
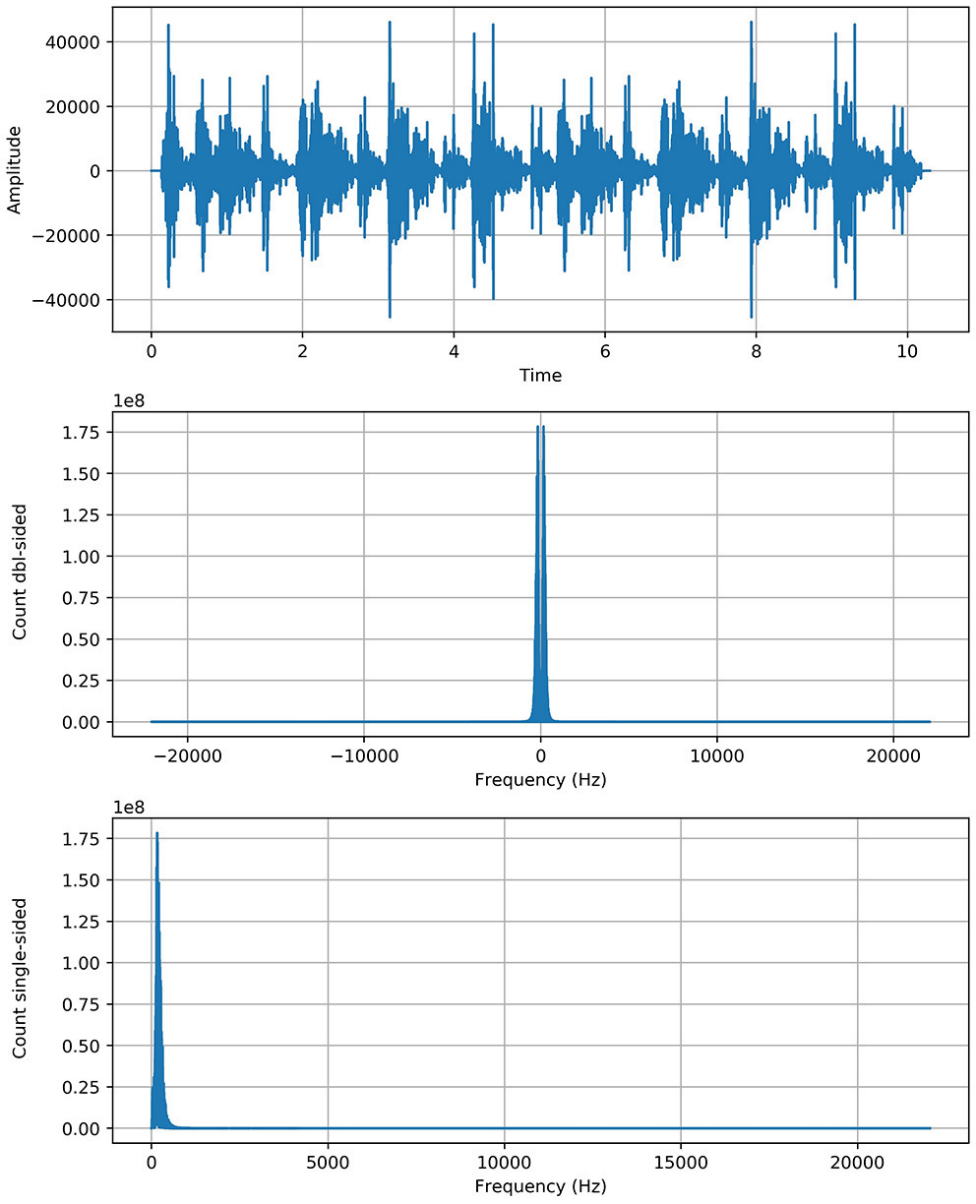
Reboot spectrogram of ARS.

As ***F***_***ft***_ represented the actual signal of RS and added twice to boost the potency/strength of a particular signal without shifting its circumstances as we see that the y-axis of the signal graph extended from 1e7 to 1e8 scale through the above equation. The Figure 1 shows the graph of each class with more strength without any alteration in genuine behavior.

### 3.5. Texture Analysis

**Figure 2:**
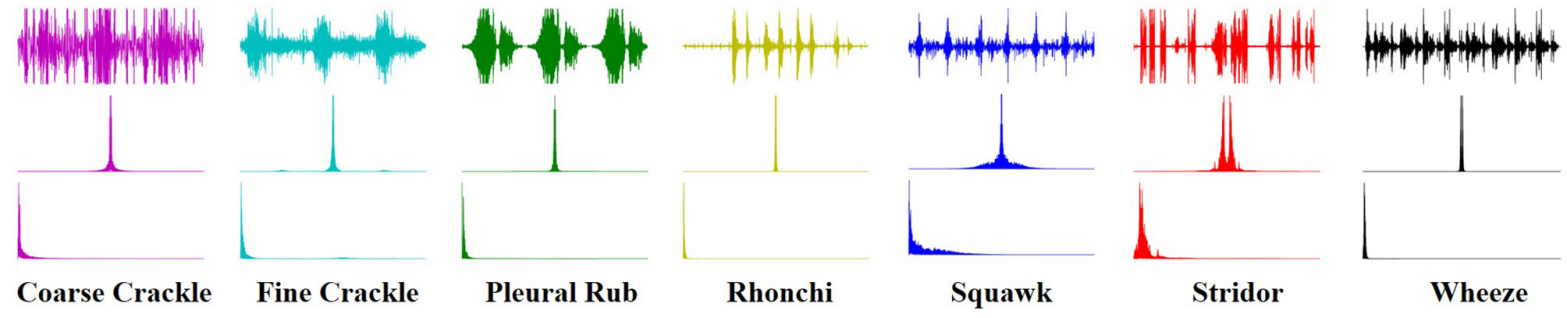
Texture analysis of ARS.

An image texture is a set of metrics calculated in spectral processing designed to quantify the perceived texture of the spectrogram. The texture of any spectrum gives us information about the spatial arrangement of color or intensities in an image or selected spectrum region. We present a set of textural measures derived from the texture spectrum. The proposed features extract textural information of an RS spectrum with complete respect to texture characteristics. Different classes of adventitious respiratory sounds get through the textural analysis and assigned different colors to enhance the performance of the features extraction in the characterization and discrimination of the texture aspects of spectrograms. Texture analysis of ARS for each target class is visualized in Figure 2.

### 3.6. Feature Extraction

Feature extraction is an attribute reduction process. Unlike feature selection, which ranks the existing attributes according to their predictive significance, feature extraction transforms them. The feature is an individual measurable property or characteristic of a phenomenon being observed. Feature extraction is an attribute reduction process; unlike feature selection, which ranks the existing attributes according to their predictive significance, FE transforms them. Choosing informative and discriminating is a crucial step for practical algorithms in pattern recognition and classification. FE is done to enhance the effectiveness of classification. In the case of respiratory sounds, feature extraction is done through feature mapping. Feature mapping was applied to extract the patterns or maps of RS features. We applied a filter on the input spectrum and got the map of the spectrum as output. The output of the feature map gave us a visual understanding of the RS feature.

### 3.7. Data Augmentation

Data augmentation techniques are used to normalize the data and increase the number of dataset elements into multiples, e.g., padding, cropping, flipping, and removing all those factors that may be considered errors. The augmentation techniques have been tested to increase the cardinality of the training set for all the classes and overcome the problem of over-fitting. One augmentation technique applied to the spectrograms and is horizontal flipping.

**Horizontal Flip:** A simple idea for data augmentation is “horizontal flipping,” which was applied on spectrograms. Each spectrogram was randomly flipped from left to right.

### 3.8. Classification

Finally, multiple classifiers were applied to check which of them gave the best results in terms of accuracy, which was considered a primary performance measure of research. Deep learning algorithms implemented such as VGG (VGG-B1, VGG-B3, VGG-V1, VGG-V2, and VGG-D1), AlexNet, ResNet, InceptionNet, and LeNet, on the spectral data for classification purposes and analyze the results and compare it with each other for better classification of abnormal respiratory sounds.

- VGG: VGG stands for Visual Geometry Group. In 2014, Simonyan and Zisserman launched VGG network architecture. Now it has numerous variants like VGG-B1, VGG-B3, VGG-V1, VGG-V2, and VGG-D1.
- AlexNet: Its convolutional neural network was developed by Alex Krizhevsky, Geoffrey Hinton, and Ilya Sutskever in 2012. Sixty million parameters are involved in its architecture.
- ResNet: often known as Residual Neural Networks designed in 2015 by Kaiming He, having 3.6% error rate.
- InceptionNet: Google designed the CNN known as GoogleNet or InceptionNet in 2014. Four million parameters were incorporated with a 6.67% error rate.
- LeNet: Develop by Yann LeCun et al. Number of parameters are 60,000 involved in processing. Error rates were also not defined because it was designed in 1998.

“AlexNet” is an effective model for attaining excessive accuracies on complicated data units. It is a leading structural design for any object recognition challenge. AlexNet consists of five convolutional layers and three fully connected layers.

- First Convolutional Layer (CL1) has 96 filters with kernel size (11x11) for extracting features for a particular spectrogram of RS. The Stride for CL1 is four for compressing the spectrogram, which moves the filter to four pixels at a time. Max-Pooling (MP1) overlapped CL1 kept padding valid the same as CL1. Filters are the same, but the kernel size is (2x2), different from CL1. As we talk about Stride, it is two for Max-pooling.
- The second Convolutional Layer (CL2) of the network has 256 filters with the same kernel size (CL1). Stride is two for compressing the spectrogram, which moves the filter to two pixels at a time. Max-Pooling (MP2) overlapped CL2 kept padding valid the same as CL2. Filters of MP2 are equal to CL2 filters. Kernel size and strides are the same as the MP1 layer.
- The third and fourth convolutional layers (CL3 & CL4) have 384 filters with the same kernel size (3 × 3). Both of them have one Stride for moving filter to 1 pixel with valid padding. Any pooling layer does not follow CL3 & CL4. The fifth Convolutional Layer (CL5) has 256 filters with the same kernel size (3 × 3). Stride is (1) for compressing the spectrogram, which moves the filter to 1 pixel at a time.
- Max-Pooling (MP3) overlapped to CL5 kept padding valid. Filters of MP3 are equal to CL5 filters. Kernel size and Stride are (2 × 2) and (2), respectively. Three fully connected layers (FCL1, FCL2, & FCL3) are involved at the end of convolutional layers, altering data in 1-dimension and finally classification performed on that single long array of data.

**Figure 3:**
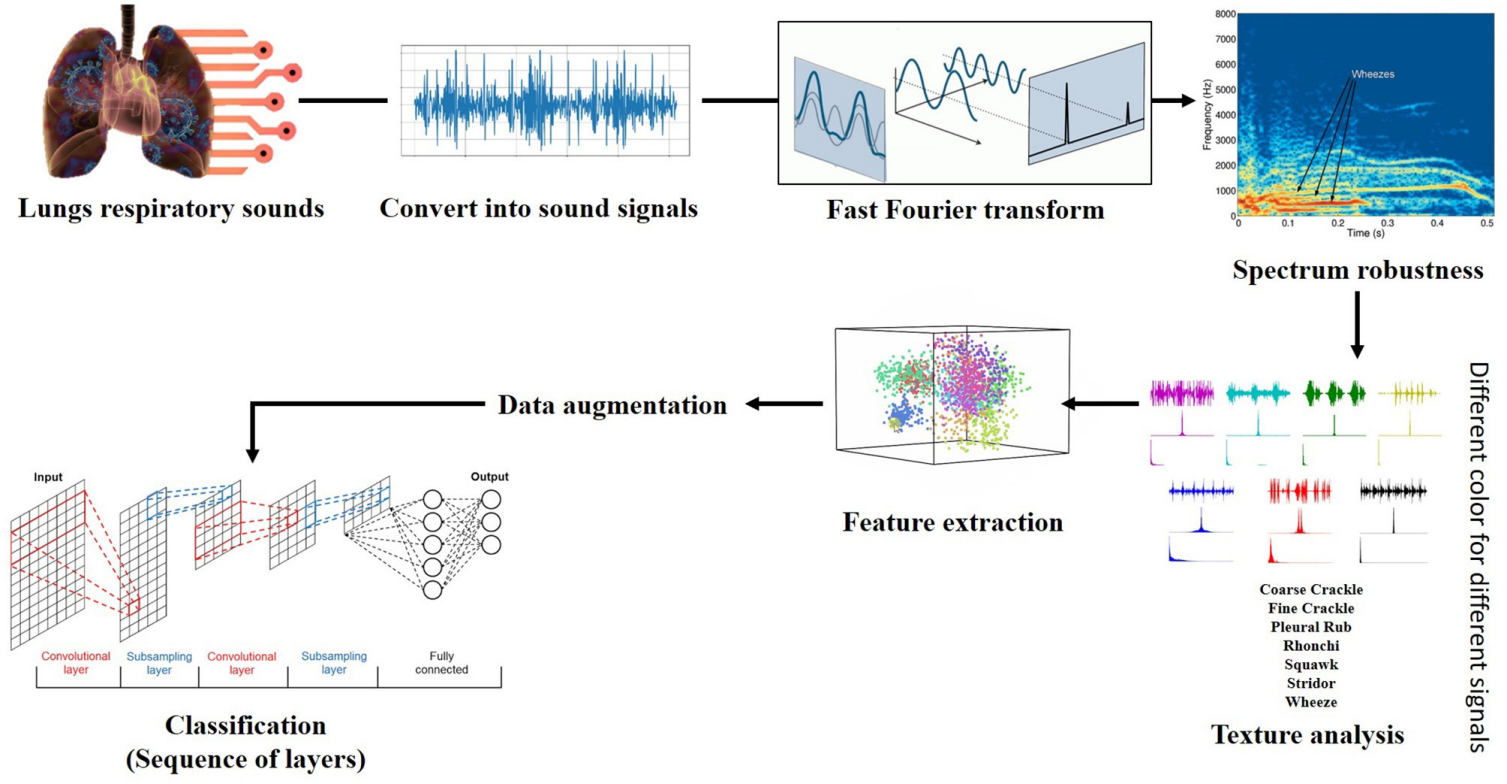
Architecture of proposed framework.

The flow chart of proposed framework is given in Figure 3.

## 4. Results and Discussion

### 4.1. Dataset Collection

Mainly researchers use datasets from multiple repositories for research purposes, and all of them were primarily generated for academic aspire. Every catalog contains a different amount of mock-ups. Unluckily, most of these repositories have limited classes of respiratory/ lung sounds. If someone wants to examine all respiratory sounds (abnormal), he/ she will not discover any solo platform from where they can get the desired/ preferred dataset.

In this research work, we assemble datasets from multiple internet sources [e.g., “(R.A.L.E. Lung Sounds 3.2),” “Thinklabs One (digital stethoscope),” and “Easy Auscultation”] used in prior research work. Founded respiratory sounds were not in symmetry, some of these sources contain hundreds of samples, and some of them hardly have few entities for particular classes. So we took a small number of entities from the above-cited foundations for purposed research. The number of target instances corresponds to each class are: Wheeze (12), Rhonchi (9), Stridor (10), Squawk (8), Fine Crackle (11), Coarse Crackle (11), and Pleural Rub (9).

### 4.2. Evaluation Metrics

We divided the dataset into two sets of classes. We train a classifier on 70% data and test on 30% to apply different classifiers to perform the analysis. The ratio of training and testing sets was decided based on random selection.

The performance of the system evaluated using Percision, Recall, F1-Score, and Accuracy.

### 4.3. Results

#### 4.3.1. VGG-B1

Table 2 shows, “VGG-B1” has a precision of 0.67 for fine crackle sounds, and recall for a plural rub is recorded as 0.50. F1-score recorded as 0.80 and 0.67 for fine crackle and plural rub, respectively. Other results of precision and recall reached a 1.00 score for all classes of ARS. As we talk about the accuracy results, the overall accuracy of VGG-B1 for all abnormal RS classes is traced as 0.95%.

**Table 2:** Comparative results obtained (Precision and Recall) for VGG-B1.

	**Precision**	**Recall**	**F1-score**
Coarse crackle	1.00	1.00	1.00
Fine crackle	0.67	1.00	0.80
Pleural rub	1.00	0.50	0.67
Rhonchi	1.00	1.00	1.00
Squawk	1.00	1.00	1.00
Stridor	1.00	1.00	1.00
Wheeze	1.00	1.00	1.00
Accuracy			0.95
Macro avg	0.95	0.93	0.92
Weighted avg	0.96	0.95	0.94

#### 4.3.2. VGG-B3

**Figure 4:**
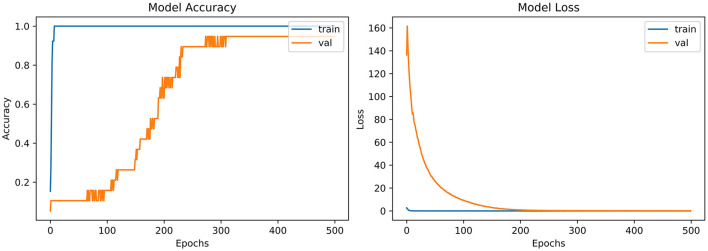
Model accuracy and model loss for VGG-B3.

From the accuracy plot of VGG-B3 (referred to Figure 4), the model was trained for 500 iterations (epoch). The training curve remains stable during iterations because it learns speedily as the training dataset given to it. On the other side, the validation/accuracy curve in the accuracy model shows that in the start, it underlines, but when the epoch is exceeded, the curve rises slowly, and in the last few epochs, it shows consistency with the training curve.

The model has the same iteration as the inaccuracy model from the loss plot of VGG-B3 (referred to Figure 4). The loss of the model is far above the ground at the starting stage of the iterations invalidation process. However, when epochs increase, a reduced amount of loss is observed compared to the earlier testing stage.

#### 4.3.3. VGG-V1

Table 3 shows that “VGG-V1” has unique and poor results compared to other algorithms. “VGG-V1” has a precision of 0.67 for coarse and fine crackle and 0.00 for the pleural rub. On the other side, the recall is recorded as 0.67, 0.00 for stridor and pleural rub, respectively. F1-score was captured as 0.80 for CC, FC, and stridor, whereas 0.00 is for the pleural rub. Other results of precision and recall hit a 1.00 score for all ARS. In VGG-V1, the overall accuracy for all abnormal RS classes is achieved as 0.84%.

**Table 3:** Comparative results obtained (Precision and Recall) for VGG-V1.

	**Precision**	**Recall**	**F1-score**
Coarse crackle	0.67	1.00	0.80
Fine crackle	0.67	1.00	0.80
Pleural rub	0.00	0.00	0.00
Rhonchi	1.00	1.00	1.00
Squawk	1.00	1.00	1.00
Stridor	1.00	0.67	0.80
Wheeze	1.00	1.00	1.00
Accuracy			0.84
Macro avg	0.76	0.81	0.77
Weighted avg	0.79	0.84	0.80

#### 4.3.4. VGG-V2

Table 4 shows that “VGG-V2” has similar results with “VGG-V1” in terms of accuracy. “VGG-V2” has a precision of 0.00 for the pleural rub. 0.75, 0.67 precision is also recorded for stridor and wheeze, respectively. The recall is recorded as 0.00 for a pleural rub and 0.50 for rhonchi. F1-score captured as 0.00 for pleural rub, 0.67 for rhonchi, 0.86 for stridor, and 0.80 for wheeze. In VGG-V2, the concerning outcome of overall accuracy for all classes is achieved as 0.84%.

**Table 4:** Comparative results obtained (Precision and Recall) for VGG-V2.

	**Precision**	**Recall**	**F1-score**
Coarse crackle	1.00	1.00	1.00
Fine crackle	1.00	1.00	1.00
Pleural rub	0.00	0.00	0.00
Rhonchi	1.00	0.50	0.67
Squawk	1.00	1.00	1.00
Stridor	0.75	1.00	0.86
Wheeze	0.67	1.00	0.80
Accuracy			0.84
Macro avg	0.77	0.79	0.76
Weighted avg	0.79	0.84	0.79

#### 4.3.5. VGG-D1

Table 5 shows that “VGG-D1” has similar results as “VGG-B1” and “VGG-B3.” VGG-D1 has a precision of 0.67 for fine crackle sounds, and recall for a pleural rub is recorded as 0.50. F1-score captured as 0.80 and 0.67 for fine crackle and plural rub, respectively. Other results of precision and recall reached a 1.00 score for all classes of ARS. While taking a look at the accuracy results, the overall accuracy of VGG-D1 for all abnormal RS classes is achieved as 0.95%.

**Table 5:** Comparative results obtained (Precision and Recall) for VGG-D1.

	**Precision**	**Recall**	**F1-score**
Coarse crackle	1.00	1.00	1.00
Fine crackle	0.67	1.00	0.80
Pleural rub	1.00	0.50	0.67
Rhonchi	1.00	1.00	1.00
Squawk	1.00	1.00	1.00
Stridor	1.00	1.00	1.00
Wheeze	1.00	1.00	1.00
Accuracy			0.95
Macro avg	0.95	0.93	0.92
Weighted avg	0.96	0.95	0.94

#### 4.3.6. AlexNet

**Figure 5:**
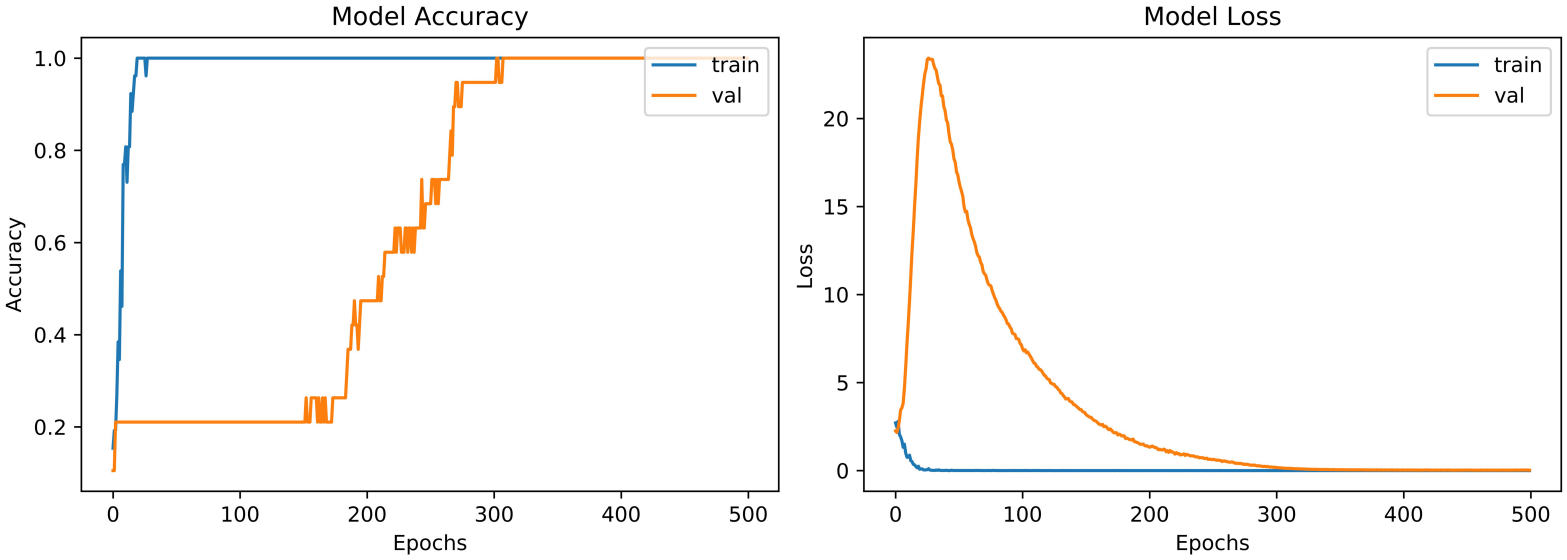
Model accuracy and model loss for AlexNet.

From the accuracy plot of AlexNet (referred to Figure 5), the model was trained for 500 iterations (epoch). At the start of the training process model, it becomes skilled fast on the training dataset, so it remains regular after further iterations. On the other hand, the validation accuracy curve in the accuracy model remains straight and contradicts the training curve to 150 epochs. After 150–300 epochs, it gradually increases and meets the training curve till the end of epochs.

From loss plot of AlexNet (referred to in Figure 5) has the same iteration as did in the accuracy model. A significant loss was observed in the model at the early stages of validation. Then suddenly, the loss reduces after 100 iterations and meets with the training curve after 300 epochs.

#### 4.3.7. InceptionNet

Table 6 shows that “InceptionNet” has a precision of 0.67 for rhonchi sounds, and recall for stridor is recorded as 0.67. F1-score was captured as 0.80 for both rhonchi and stridor. Other results of precision and recall reached a 1.00 score for all classes of ARS. While looking at the accuracy results, the overall accuracy of “InceptionNet” for all abnormal RS classes is achieved as 0.95%.

**Table 6:** Comparative results obtained (Precision and Recall) for InceptionNet.

	**Precision**	**Recall**	**F1-score**
Coarse crackle	1.00	1.00	1.00
Fine crackle	1.00	1.00	1.00
Pleural rub	1.00	1.00	1.00
Rhonchi	0.67	1.00	0.80
Squawk	1.00	1.00	1.00
Stridor	1.00	0.67	0.80
Wheeze	1.00	1.00	1.00
Accuracy			0.95
Macro avg	0.95	0.95	0.94
Weighted avg	0.96	0.95	0.95

#### 4.3.8. LeNet-5

**Figure 6:**
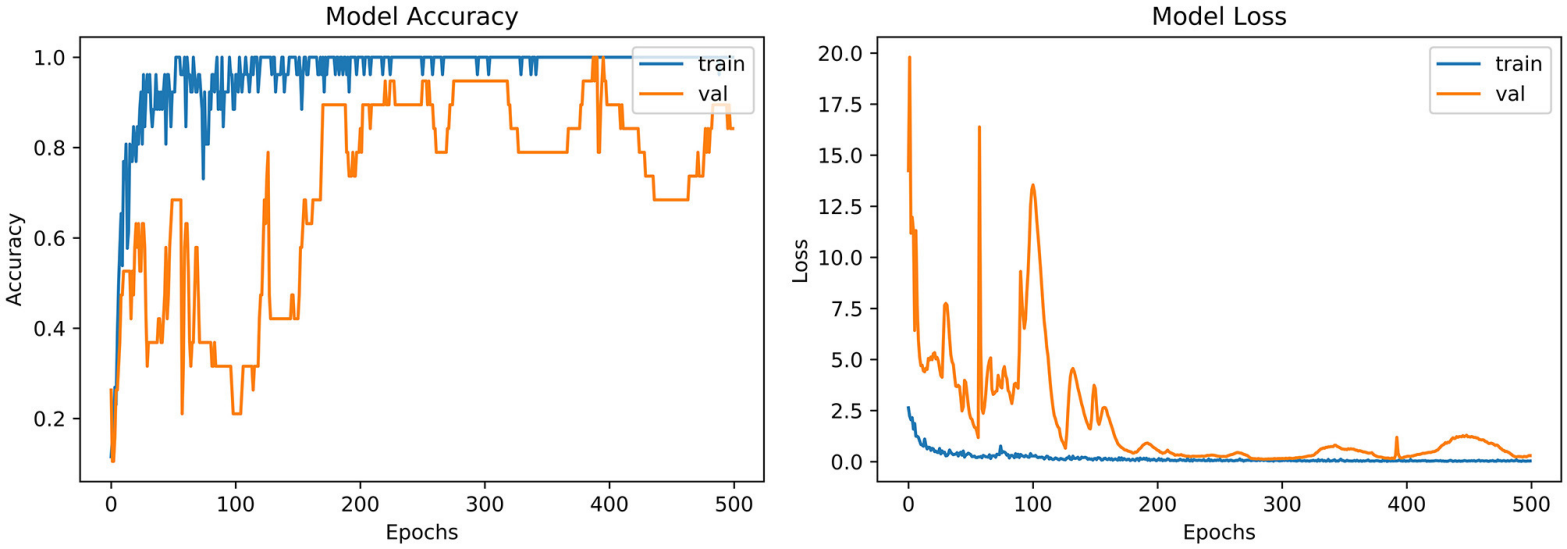
Model accuracy and model loss for LeNet-5.

From the accuracy plot of LeNet-5 (referred to Figure 6), the model was trained for 500 iterations (epoch). At the early stages of the process, the model shows high inflection in many curve points and rises with unrepresentative manners. It shows a steady curve after 350 iterations of training. On the other side, the Val-acc curve shows a considerable divergence from the start to the end of the model. Val-curve rising in random manners from the start of the model and shows vulnerability in behavior.

The loss plot of LeNet-5 (referred to Figure 6) has the same iteration as did in the accuracy model. The validation curve shows “unrepresentative” in the model. High loss between training and validation curves at the starting stage was observed. When iterations were performed in the testing process, loss reduces in contrast with the earlier validation stage.

#### 4.3.9. ResNet

**Figure 7:**
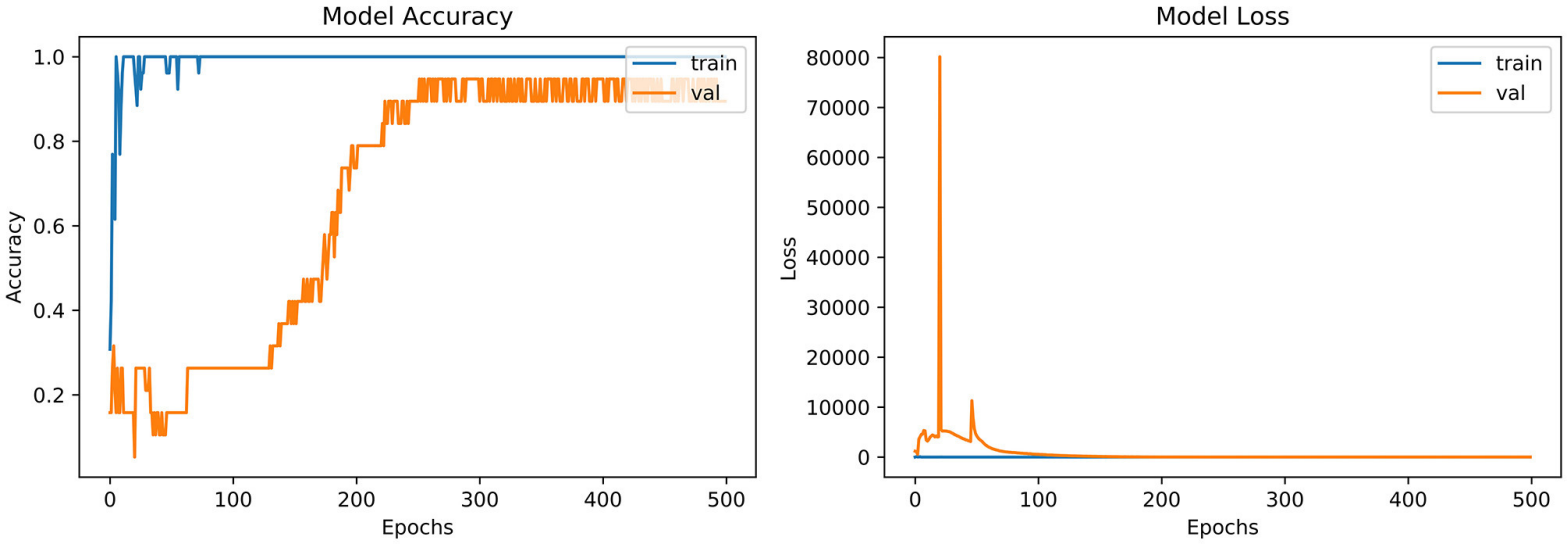
Model accuracy and model loss for ResNet.

From the accuracy plot of ResNet (referred to Figure 7), the model was trained for 500 iterations (epoch). At the start of the training, the process model is trained swiftly, showing a steady curve during further iterations for training. Before obtaining steadiness, it shows the variation in multiple points. While seeing at the Val-acc curve, the significant deviation was noticed, but after few epochs, it suddenly arises to meet the training curve. Then again Val-acc curve became steady from 200 epochs and onward.

From the loss plot of ResNet (referred to Figure 7), the model shows deviation and high loss between training and validation curves at the early stage. However, after 100 epochs, the reduced amount of loss was observed in contrast with an earlier validation stage, and the Val curve meets with the training curve.

### 4.4. Discussion

“VGG-B1,” “VGG-B3,” “VGG-D1,” and “ResNet” have similar results in terms of accuracy which is recorded as 0.95%. Figure 4 shows similar curves as the model accuracy curve shows that model trained fits well. The validation curve is a little dissatisfactory due to underfitting, which enhances the model's training. In the model loss, the curve outcome represents that training reduces the loss. In Figure 7, the accuracy model curve indicates that the model could be trained more to avoid the underfitting and inflection in some points because the model has not been overlearned for the training set. Model loss shows a divergence from the training curve due to less training, which is why the loss is high from starting epochs.

Accuracy is captured as 0.84% for VGG-V1 and VGG-V2 (Tables 3, 4), which is relatively low compared to other classifiers. The reason is that both classifiers need more iteration or data samples for training to enhance the accuracy.

Refer to Table 6, which represent that InceptionNet has accuracy of 0.95%. Figure 6 point toward the accuracy model curve indicates that the model can be trained further to avoid the underfitting and dissimilarity between training and validation because the model has not learned enough for the validation set. Model loss shows a divergence from the training curve due to less training, which is why the loss is high from the start to the end of epochs.

### 4.5. Comparative Analysis

For comparison, the analysis was performed based on mean precision, recall, f1-score, and accuracy for all algorithms. Overall results from the classifiers used for ARS classification are shown in Table 7.

**Table 7:** Comparative results obtained (Precision, Recall, F1-score, and Accuracy) for all algorithms employed.

	**Precision**	**Recall**	**F1-score**	**Accuracy (%)**
VGG-B1	0.95	0.93	0.92	0.95
VGG-B3	0.95	0.93	0.92	0.95
VGG_Drop	0.95	0.93	0.92	0.95
VGG-V1	0.76	0.81	0.77	0.84
VGG-V2	0.77	0.79	0.76	0.84
AlexNet	1.00	1.00	1.00	1.00
InceptionNet	0.95	0.95	0.94	0.95
ResNet	0.95	0.93	0.92	0.95
LeNet5	0.95	0.90	0.90	0.89

**Figure 8:**
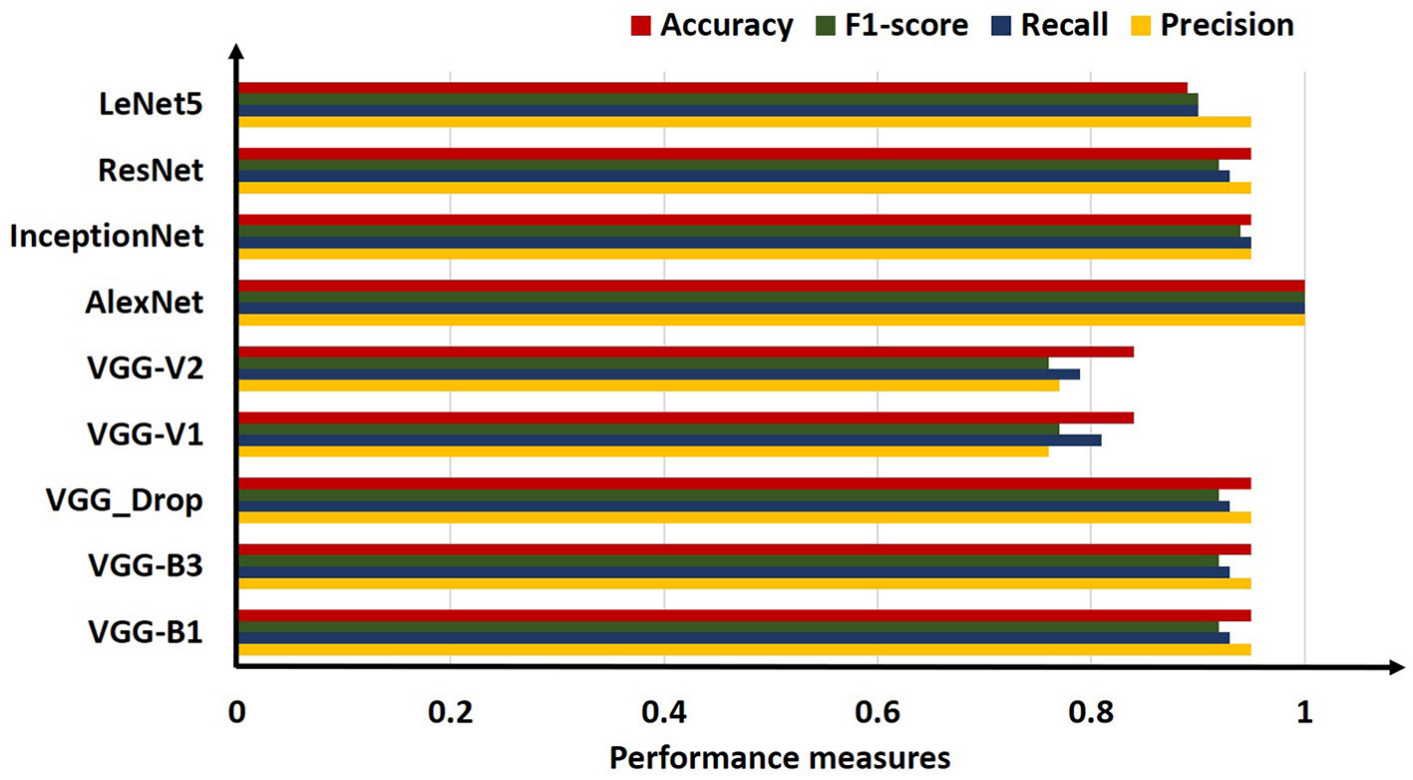
Comparative bar chart visualization obtained for all algorithms employed.

The above table shows that the “AlexNet” algorithm has the highest accuracy rate than other classifiers. 1.00% classification accuracy is captured for AlexNet, other algorithms mostly have 0.95% accuracy, and some have below 0.95%. Graphical representation of the above results is as following in Figure 8.

## 5. Conclusion

The possible concern with the neural network approach is that barely a few units can be employed for classification. If further research is conducted on this domain, researchers can consider more sounds or build their dataset for the classification task, especially for COVID-19. Researchers may integrate more features such as sound quality (timbre) for classification in a new-fangled way, train the neural network for a healthier consequence, and upgrade the classification scheme. RS attributes and their analysis gives symptomatic data about a patient's lung. A couple of decades back, doctors distinguish symptomatic signs in lung sounds through a typical stethoscope, usually considered as a cheap and secure method for examining. Lung diseases are the third most common cause of death worldwide, so it is essential to classify RS abnormality to overcome the death rate accurately.

In this research, ANA methods are used in conjunction with different deep convolutional networks to classify the various abnormal respiratory sounds—both continuous and discontinuous. Visual inspection of abnormal respiratory sound was done by Fourier analysis. The presence of abnormal sounds like a wheeze, stridor, fine crackle, and coarse crackle was revealed when Fourier Transform was applied over a short time duration and frequency. Texture analysis was performed for better feature extraction through feature maps and data augmentation executed for enlarging the number of entities. Numerous algorithms were applied on a range of spectrograms, and results obtained from this method were satisfactory as it considered the seven classes of abnormal RS concurrently 1.00% accuracy gained through the AlexNet algorithm.

## Data Availability Statement

The original contributions presented in the study are included in the article/supplementary material, further inquiries can be directed to the corresponding author/s.

## Author Contributions

All authors listed have made a substantial, direct and intellectual contribution to the work, and approved it for publication.

## Funding

This work was supported by the United Arab Emirates University (UAEU Grant No. G00003270 31T130).

## Conflict of Interest

The authors declare that the research was conducted in the absence of any commercial or financial relationships that could be construed as a potential conflict of interest.

## Publisher's Note

All claims expressed in this article are solely those of the authors and do not necessarily represent those of their affiliated organizations, or those of the publisher, the editors and the reviewers. Any product that may be evaluated in this article, or claim that may be made by its manufacturer, is not guaranteed or endorsed by the publisher.
