# Corrigendum: Abnormal respiratory sounds classification using deep CNN through artificial noise addition

**DOI:** 10.3389/fmed.2024.1545847

**Published:** 2025-01-07

**Authors:** Rizwana Zulfiqar, Fiaz Majeed, Rizwana Irfan, Hafiz Tayyab Rauf, Elhadj Benkhelifa, Abdelkader Nasreddine Belkacem

**Affiliations:** ^1^Faculty of Computing and Information Technology, University of Gujrat, Gujrat, Pakistan; ^2^Department of Information Technology, College of Computing and Information Technology at Khulais, University of Jeddah, Jeddah, Saudi Arabia; ^3^Independent Researcher, Bradford, United Kingdom; ^4^Cloud Computing and Applications Reseach Lab, Staffordshire University, Stoke-on-Trent, United Kingdom; ^5^Department of Computer and Network Engineering, College of Information Technology, UAE University, Al Ain, United Arab Emirates

**Keywords:** respiratory sounds, abnormal respiratory sounds, continuous adventitious sounds (CAS), discontinuous adventitious sounds (DAS), deep CNN

In the published article, there was an error in affiliation(s) [3]. Instead of “[Centre for Smart Systems, AI and Cybersecurity, Staffordshire University, Stoke-on-Trent, United Kingdom]”, it should be “[Independent Researcher, Bradford, United Kingdom]”.

The authors apologize for this error and state that this does not change the scientific conclusions of the article in any way. The original article has been updated.

